# Identification and management of subclinical disease activity in early multiple sclerosis: a review

**DOI:** 10.1007/s00415-023-12021-5

**Published:** 2023-10-21

**Authors:** Daniel Ontaneda, Tanuja Chitnis, Kottil Rammohan, Ahmed Z. Obeidat

**Affiliations:** 1 Mellen Center for Multiple Sclerosis, Department of Neurology, Cleveland Clinic, Cleveland, OH USA; 2 Brigham Multiple Sclerosis Center, Brigham and Women’s Hospital, Harvard Medical School, Boston, MA USA; 3 Division of Multiple Sclerosis, University of Miami Miller School of Medicine, Miami, FL USA; 4 Department of Neurology, Medical College of Wisconsin, Milwaukee, WI USA

**Keywords:** Biomarkers, Cognition, Disease activity, Fatigue, High-efficacy therapy, Multiple sclerosis

## Abstract

**Importance:**

Early treatment initiation in multiple sclerosis (MS) is crucial in preventing irreversible neurological damage and disability progression. The current assessment of disease activity relies on relapse rates and magnetic resonance imaging (MRI) lesion activity, but inclusion of other early, often “hidden,” indicators of disease activity may describe a more comprehensive picture of MS.

**Observations:**

Early indicators of MS disease activity other than relapses and MRI activity, such as cognitive impairment, brain atrophy, and fatigue, are not typically captured by routine disease monitoring. Furthermore, silent progression (neurological decline not clearly captured by standard methods) may occur undetected by relapse and MRI lesion activity monitoring. Consequently, patients considered to have no disease activity actually may have worsening disease, suggesting a need to revise MS management strategies with respect to timely initiation and escalation of disease-modifying therapy (DMT). Traditionally, first-line MS treatment starts with low- or moderate-efficacy therapies, before escalating to high-efficacy therapies (HETs) after evidence of breakthrough disease activity. However, multiple observational studies have shown that early initiation of HETs can prevent or reduce disability progression. Ongoing randomized clinical trials are comparing escalation and early HET approaches.

**Conclusions and relevance:**

There is an urgent need to reassess how MS disease activity and worsening are measured. A greater awareness of “hidden” indicators, potentially combined with biomarkers to reveal silent disease activity and neurodegeneration underlying MS, would provide a more complete picture of MS and allow for timely therapeutic intervention with HET or switching DMTs to address suboptimal treatment responses.

## Introduction

Multiple sclerosis (MS) is a chronic, heterogenous, inflammatory and neurogenerative disease, characterized by demyelinating lesions in the central nervous system. MS can be categorized as clinically isolated syndrome (CIS), relapsing–remitting MS (RRMS), primary progressive MS, and secondary progressive MS (SPMS) [1]. An early asymptomatic phase with brain magnetic resonance imaging (MRI) abnormalities suggestive of MS has been termed radiologically isolated syndrome (RIS) [2].

Effective control of MS disease activity initiated early in the disease course, before MS-related nervous system damage becomes irreversible, is critical. Early and accurate MS diagnosis allows for early intervention to optimize long-term patient outcomes [3, 4]. This is particularly relevant in children and younger adults in whom treatment effects are amplified and have greater success in slowing progression, when compared with older patients [5, 6].

Currently, clinical guidelines define MS activity as demonstration of relapses or radiologic activity, which are measured by new or active lesions on MRI [7, 8]. However, relapses and MRI lesions largely reflect only the focal inflammatory aspects of disease activity; the accrual of disability, manifested through worsening on neurological examination, and more subtle signs such as fatigue or impaired cognition, may be missed. Furthermore, an apparent clinical–radiologic paradox exists, where clinical and radiologic evidence can be poorly correlated [9]. Advanced imaging, nonimaging and soluble biomarkers, such as neurofilament light chain (NfL) levels, may provide more sensitive measures of underlying disease activity that could help explain the clinical–radiologic paradox. Measures of disease activity that capture inflammatory, neurodegenerative, and disability elements may allow faster identification of underlying disease activity and suboptimal response to therapy, thus improving MS risk assessment and treatment decision making.

The implementation of high-efficacy therapies (HETs) as first-line treatment may delay disability progression and improve clinical outcomes [3, 10]. However, the current guidelines recommend HET use in patients with highly active MS [7, 11, 12]. Moreover, definitions of highly active disease have differed across clinical trials and include the occurrence of clinical relapses and lesion activity as detected by MRI [7], which may not fully capture disease activity.

This review explores early, often “hidden” indicators of disease activity, such as changes in cognition and fatigue and biomarkers of disease activity, to support characterization of a more comprehensive picture of MS, and which should be considered in the management of confirmed early MS. We also present current evidence regarding the early use of HETs and the unmet need for treatment guidelines that include early MS activity and recommendations regarding prompt HET intervention.

## Current definitions of disease activity

### MS diagnosis

MS is diagnosed according to the most recent version of the McDonald criteria [13]. This requires confirmation of central nervous system (CNS) disease disseminated in time and space as demonstrated by clinical attacks, examination features, MRI, and cerebrospinal fluid (CSF) analysis [13]. The McDonald criteria and its subsequent revisions have helped to decrease delays in diagnosis, notably among children, adolescents, and younger adults [14–16]. Clinical relapses and MRI disease activity are prioritized in the most recent criteria [13].

Although the 2017 updates to the McDonald criteria aimed to provide guidance to avoid the misdiagnosis of MS [13], it is important to acknowledge that misdiagnosis remains a concern in clinical practice [17, 18]. One 2019 study found that approximately 1 in 5 patients with a diagnosis of MS did not actually meet the diagnosis criteria upon reevaluation at an MS subspecialty center [17]. The proper application of the McDonald criteria is critical for a correct diagnosis of MS, as patients receiving a misdiagnosis of MS may be exposed to unnecessary risks when incorrectly prescribed DMTs and may incur considerable financial burden [17].

### Disease activity in clinical guidelines

Practice guidelines from the American Academy of Neurology measure disease activity by clinical relapses or new MRI lesions, and these assessments are used to guide MS monitoring and treatment [7]. Similarly, treatment guidelines from the European Committee of Treatment and Research in MS and the European Academy of Neurology describe disease activity in patients with relapsing forms of MS in terms of relapses, disability progression, and MRI activity [11].

## Early disability accrual in MS

**Fig. 1:**
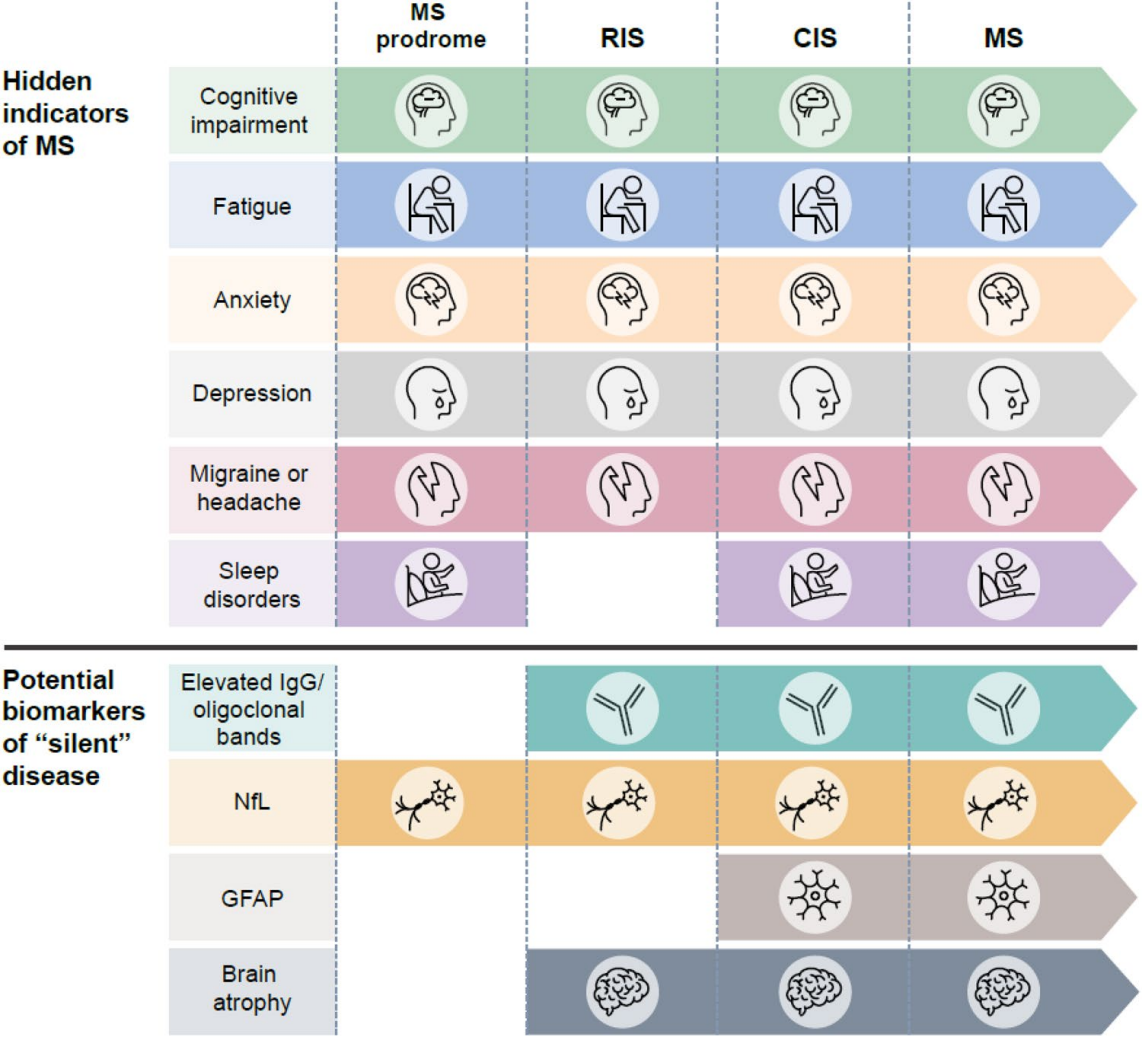
Subclinical or “hidden” indicators of disease activity, identified from prediagnosis of MS through early RRMS, that represent disease activity. *CIS*, clinically isolated syndrome, *GFAP* glial fibrillary acidic protein, *IgG*, immunoglobulin G, *MS* multiple sclerosis, *NfL* neurofilament light, *RIS* radiologically isolated syndrome, *RRMS* relapsing–remitting multiple sclerosis

There is considerable evidence that, in the early stages of MS, pathological changes occur that are not reflected in relapses and MRI lesions. “Hidden” pathological changes such as decreased cognitive performance, anxiety, depression, and migraine, have been detected years before typical MS symptoms appear and have been identified as prodromal features in MS (Fig. 1) [19–40]. In addition, the disease progression can occur independently of relapses in patients with RRMS, termed progression independent of relapse activity (PIRA) [41, 42]. In phase 3 trials in recently diagnosed treatment-naïve patients with relapsing MS, over half of confirmed disability worsening events occurred due to PIRA [43].

### Impaired cognition

Cognitive impairment is a core symptom of MS; patients consider nonphysical MS symptoms such as cognitive impairment as having a substantial impact on their quality of life [30, 44]. Guidelines for identifying and monitoring cognitive changes in MS were published in 2018 by the National Multiple Sclerosis Society, but do not discuss prognostic applications [30].

Cognitive impairment has been detected in patients with RIS and may be consistent with a diagnosis of subclinical MS [24]; cognitive impairment in CIS may actually predict conversion to MS [45]. Cognitive impairment can be common in early MS [40, 46]: in a study of 92 patients with RRMS with very mild or no clinical disability, half had cognitive impairment (across five tools measuring verbal and visuospatial long-term memory, information processing speed, and executive functions) [47]. Conversely, a study in patients with RRMS (*N* = 128) found impairments in phonemic fluency in patients with early RRMS (< 3 years) versus healthy controls (*N* = 63), but other assessments did not differ [48]. Regarding children with MS, there is disagreement on whether changes in academic performance indicate MS prodromal cognitive decline; one study identified a potential link [49], whereas another did not [50].

The importance of detecting these early cognitive changes is apparent from a study in > 1000 patients with MS that showed irreversible axonal damage and brain tissue loss accumulation before cognitive decline became evident [51]. Cognitive impairment has also been linked to CSF molecular patterns showing innate and adaptive immune responses in newly diagnosed patients [52] and gray matter damage [53]. Although cognitive impairment may go unnoticed in early MS due to compensatory neurological mechanisms [54], early control of inflammation could prevent or limit cognitive impairment. Moreover, changes in cognition could indicate suboptimal MS disease control and underlying disease activity and progression and therefore signify an urgency to initiate or change MS treatment [30, 45].

A unified set of criteria for identifying cognitive impairment early in MS is needed [55]. Several validated assessments already exist and are summarized in Table 1 [30, 56–58]. Because no single tool is currently widely used, it is reasonable to adopt an easy-to-use tool not requiring psychological training to administer in clinical practice. However, such a tool should be validated to the larger, more complete neuropsychological battery to establish if the results are valid and if it is a useful surrogate for the more time-consuming neuropsychological battery. Additional concerns include learning effects on repeated administration, and the usefulness of repeated testing to detect cognitive decline using the same instrument has been questioned [59].

**Table 1:** Examples of assessments for cognitive impairment in MS

Assessment	Details	Disadvantages
SDMT [30, 56]	Recommended as the best rapid assessment tool for cognitive processing speed in adults in clinical practice Recommended as a cognitive measure in clinical trials No neuropsychological training required to administer test Takes around 5 min to complete	A screening tool that does not provide information about all cognitive domains
PST [30, 58]	Digital adaption of SDMT Self-administered iPad-based tool for routine use in MS clinic Capable of seamless integration with medical records Takes around 5 min to complete	A screening tool that does not provide information about all cognitive domains
BICAMS [30, 56, 57]	Includes three tests: SDMT California Verbal Learning Test–Second Edition Brief Visuospatial Memory Test–Revised Widely validated across numerous languages and cultures Can be administered in 15–20 min by health personnel without specific training	Upper limb functionality can affect its outcomes
Minimal Assessment of Cognitive Function in MS [30, 56]	Includes seven tests: SDMT Paced Auditory Serial Addition Test California Verbal Learning Test–Second Edition Brief Visuospatial Memory Test–Revised Controlled Oral Word Association Test Judgement of Line Orientation Test Delias–Kaplan Executive Function System Scoring Test Evaluates all cognitive functions affected by MS	Takes around 90 min to complete Requires clinical neuropsychology training to administer
Brief Repeatable Neuropsychological Battery [30, 56]	Includes five tests: Paced Auditory Serial Addition Test SDMT Selective reminding test 10/36 Spatial recall test Oral Work association test High sensitivity and specificity	Takes around 45 min to complete Requires clinical neuropsychology training to administer and interpret data Some cognitive functions are poorly evaluated or excluded

*BICAMS* Brief International Cognitive Assessment for MS, *MS* multiple sclerosis, *PST* Processing Speed Test, *SDMT* Symbol Digit Modalities Test

### Neuropsychiatric symptoms

Neuropsychiatric symptoms, including mild depressive symptoms and fatigue, have been reported in patients with CIS who did not meet the diagnostic MRI criteria for MS; anxiety and depression were associated with lower normal white matter volume and higher lesion load on MRI, respectively [28]. Patients can also experience sleep disorders, anxiety, depression, and migraine both before their MS diagnosis and after MS is diagnosed [3, 21, 34, 36, 38, 60, 61].

In patients with MS, fatigue is among the most common symptoms and substantially impacts quality of life [3]. Fatigue and depression are linked to cognitive impairment; fatigue has also been associated with brain lesions and both CNS and peripheral inflammation [62, 63]. Therefore, fatigue may be associated with underlying neurodegeneration or inflammation.

Currently, symptoms such as fatigue are not well captured by the Expanded Disability Status Scale (EDSS) [3], nor in the current definitions of disease activity. In addition, neuropsychiatric signs and symptoms can be the initial presenting complaint ahead of a definitive MS diagnosis [64], but these are not captured in the McDonald criteria [65, 66]. A retrospective study of 281 referrals to an MS center for a question of MS diagnosis reported that patients with only vague, nonspecific symptoms (such as headache) were not diagnosed with MS [65]. Another study reported that 96 of 244 referrals for a new diagnosis of MS presented with atypical symptoms, of whom only 15 were diagnosed with MS or CIS [67]. As such, patients exhibiting these symptoms currently have no pathway to reach a diagnosis until a clinical attack occurs or there is clear progression of neurological disability. Nonetheless, the Beck Depression Inventory—Fast Screen, the Hospital Anxiety and Depression Scale, and the Patient Health Questionnaire-9 are suggested for evaluating depression and anxiety in adults with MS [30, 68]. Monitoring children with MS for behavioral and academic performance changes is also recommended [30].

As the clinical significance of changes in these symptoms during early MS becomes more defined, updated disease management strategies may be required. For instance, clinicians’ discussions with patients and their caregivers provide opportunities to identify the first signs of “hidden” symptoms, thereby enabling earlier intervention.

## Biomarkers as indicators of “silent” disease

Although there is currently no laboratory test to diagnose MS in the absence of clinical or imaging findings [13], several molecules are elevated in the blood or CSF of patients with MS that could have the potential to be prognostic markers. However, all biomarkers described in the following sections are still in investigational stages.

### Molecular markers: focus on immunoglobulins

Oligoclonal bands (OCBs) of immunoglobulin G (IgG) are present in the CSF in up to 90% of patients with MS [26]. The presence of OCBs early in the MS disease course correlates with relapses and is associated with disease progression [26, 69, 70]. Although OCBs already form part of the McDonald diagnostic criteria [13], further confirmatory studies are required to establish OCB as a prognostic marker [71]. The IgG index is widely used as a diagnostic marker of MS and is based on elevated IgG levels in the CSF relative to the reference protein albumin; however, it can lack the sensitivity of OCBs and be influenced by age and fluctuations in albumin levels [71, 72].

### Molecular markers: focus on NfL

Multiple studies have reported elevated NfL levels in the peripheral blood and CSF of patients with MS [35, 73]. Neurofilaments are neuron-specific cytoskeletal proteins located within myelinated CNS and peripheral nervous system neurons that are released into the surrounding milieu upon neuronal damage [74, 75] (e.g., as a result of acute inflammation-mediated axonal damage [76]). NfL has been evaluated extensively in MS (Table 2) [20, 73, 77–83] and shows great potential as a biomarker in monitoring disease activity in MS [73].

**Table 2:** Examples of studies investigating NfL and GFAP as biomarkers in MS

Study	Design	Patients	Outcomes	Conclusions
NfL				
Salzer et al. (2010) [85]	Observational retrospective study	RRMS (*N* = 95)	There was correlation between CSF NfL levels and both MS severity scores and conversion to SPMS	NfL levels could be prognostic markers in early RRMS
Chitnis et al. (2018) [77]	Observational retrospective study	RRMS (*N* = 122)	Serum NfL levels were associated with 10-year MRI measures of disease worsening (T2 lesion volume and BPF)	NfL levels could be prognostic of later MRI outcomes
Bjornevik et al. (2020) [20]	Nested case − control study	MS (*N* = 30)	Serum NfL levels increased a median of 6 years before clinical symptoms appeared	Neurodegeneration was already occurring before MS clinical onset
Gaetani et al. (2019) [79]	Observational retrospective study	CIS or possible MS (*N* = 32)	CSF NfL levels were elevated at first demyelinating event before MS disease activity	CSF NfL levels are prognostic markers for very early MS A CSF NfL level cutoff of 500 pg/mL could identify patients with subsequent disease activity
Cai et al. (2018) [73]	Meta-analysis of 15 studies	Overall: 795 with CSF samples, 1856 with blood samples	Significantly higher CSF and blood NfL levels with MS vs. controls	NfL levels could be prognostic biomarkers to monitor disease activity
Bhan et al. (2021) [80]	Observational prospective study	Newly diagnosed MS (*N* = 42)	Higher CSF NfL level predicted a higher rate of atrophy and EDSS score worsening over a 10-year period	CSF NfL levels can provide indication of future disease burden up to 10 years in advance
Srpova et al. (2021) [81]	Observational prospective study	Early MS (*N* = 172)	Strong association between serum NfL levels and lesion accumulation over time; NfL levels reflected delayed BVL	NfL levels have a strong association with development of future brain atrophy
Benkert et al. (2022) [82]	Retrospective modeling and validation study	MS (*N* = 1313)	Serum NfL levels were associated with clinical and MRI measures of disease worsening; NfL levels were reduced with HET	NfL levels can be prognostic biomarkers for monitoring treatment efficacy in individual patients
Ziemssen et al. (2022) [83]	Analysis from pooled phase 3 trials	Patients with RMS, including newly diagnosed and treatment-naïve patients (*N* = 1746)	High baseline serum NfL was prognostic of accumulation of MRI lesions, higher change in WM volume, and whole brain atrophy vs. low baseline serum NfL	NfL levels are prognostic indicators of tissue damage, and potential predictors of higher probability of clinical progression
GFAP				
Momtazmanesh et al. (2021) [35]	Systematic review/meta-analysis	MS (*N* = 4071)	GFAP levels were elevated with MS vs. controls, and with PMS vs. RRMS	GFAP levels may have utility in differentiating RRMS and PMS
Martínez et al. (2015) [32]	Prospective observational study	MS (*N* = 301)	High GFAP levels were independently associated with earlier progression of EDSS score	High GFAP levels are associated with earlier disability progression
Saraste et al. (2021) [88]	Observational study	RRMS (*N* = 39), SPMS (*N* = 29)	GFAP levels were elevated in patients with MS vs. controls; high GFAP levels were correlated with T2 lesion volume, microstructural changes in brain WM, worse EDSS scores, and longer disease duration	GFAP levels could be biomarkers for MS-associated astrocytopathy and WM damage
Abdelhak et al. (2018) [151]	Prospective observational study	RRMS (*N* = 42), PMS (*N* = 38)	GFAP levels were correlated with EDSS score in patients with PMS; GFAP levels were correlated with NfL levels	GFAP levels could be disease severity markers

*BPF* brain parenchymal fraction, *BVL* brain volume loss, *CIS* clinically isolated syndrome, *CSF* cerebrospinal fluid, *EDSS* Expanded Disability Status Scale, *GFAP* glial fibrillary acidic protein, *HET* high-efficacy therapy, *MRI* magnetic resonance imaging, *MS* multiple sclerosis, *NfL* neurofilament light chain, *PMS* progressive multiple sclerosis, *RRMS* relapsing–remitting multiple sclerosis, *SPMS* secondary progressive multiple sclerosis, *WM* white matter

The 2021 Consortium of Multiple Sclerosis Centers (CMSC) Consensus Statement on Neurofilament Biomarkers in MS highlighted potential applications for NfL measurements for clinical decision-making during the course of MS, adding that serum and/or CSF NfL could complement MRI monitoring for detecting underlying inflammatory activity and informing risk of future MS disease burden [84].

There is a growing body of evidence to support NfL as a prognostic marker in early MS because it can predict MRI lesions, brain atrophy, and disability progression up to 10 years in advance (Table 2) [79–83, 85]. However, unresolved questions around cutoff values for NfL concentration and standardization of NfL measurement currently preclude its use as a biomarker in clinical practice [86]. In addition, the prognostic value of NfL level in progressive forms of MS is less clear due to the current knowledge gaps [84].

Understanding the confounding factors that can affect NfL levels, such as age, obesity, diabetes, kidney function, and certain types of drugs [84, 87], as well as assay standardization, will facilitate clinical implementation of this biomarker [84]. In addition, the use of NfL for diagnosis in real-world clinical practice requires normative NfL data with which to interpret baseline serum levels and to define clinically meaningful changes [84].

### Molecular markers: focus on glial fibrillary acidic protein (GFAP)

GFAP levels are elevated in the CSF of patients with MS [35]. GFAP has been explored as a marker of astrocyte damage and loss, which could predict disease severity, progression, and activity in MS (Table 2) [32, 88]. However, to date, there is no published evidence supporting GFAP as a marker for subclinical worsening in early MS. In addition, some of the limitations of NfL in real-world clinical practice likely apply to GFAP; for example, confounding factors affecting serum GFAP levels need to be identified and normative GFAP data are required before this marker can be used in clinical practice.

### Other molecular markers

Other molecules elevated in patients with MS include total tau protein (t-tau), chitinase-3-like protein 1 (CHI3L1), and S100B; t-tau and CHI3L1 are also elevated in those with CIS [35]. These biomarkers could potentially be used in the diagnosis and/or to prognosticate risk of MS. A meta-analysis in 338 patients reported decreased levels of brain-derived neurotrophic factor (BDNF) levels in the blood of patients with MS [89]. BDNF levels in the CSF at the time of MS diagnosis were inversely associated with cognitive performance in one study; therefore, the authors proposed that BDNF in combination with NfL could be a potential biomarker for impaired cognition in MS but recognize that integration of these measurements into clinical practice could be challenging [90]. Further research is needed to fully understand the role of these markers in the disease course [35, 90].

### MRI-based biomarkers in early MS

Brain atrophy as detected by MRI is estimated to occur at ~ 0.1–0.3% per annum as part of the normal aging process; however, age-dependent atrophy occurs more rapidly at an annual rate of 0.5–1.3% in untreated MS [91, 92]. The neurologic reserve represents the capacity of the CNS to compensate for injury through remodeling [3]; it has been proposed that brain atrophy in patients with subclinical MS depletes the neurologic reserve to a point where the brain can no longer compensate for MS-related damage, after which clinical symptoms become apparent and the disease progresses [3]. In fact, a cohort study in 140 patients with MS revealed that the rate of brain atrophy was highest during the first 5 years of the disease, especially in younger patients [93]; furthermore, early brain atrophy may be associated with early focal lesion accumulation [94] and with higher fatigue and cognitive impairment [95]. Although the tools for measuring brain atrophy at the patient level in clinical practice have been recently described, longitudinal studies to assess brain atrophy changes over time are required to test the reliability of these MRI tools [95]. Consistency between clinical MRI scans over time can also be difficult to achieve [96]. Nevertheless, early treatment with DMTs could prevent this accelerated damage to the CNS [3].

Paramagnetic rim lesions (PRLs), also known as iron rim lesions (IRLs), are thought to reflect chronic active lesions with substantial microglia/macrophage inflammation [97, 98]. Retrospective studies of patients with MS have found that, compared with patients without PRLs, patients with least one PRL had higher disability scores, T2 lesion volume, and intrathecal IgG synthesis were higher, and lower brain volume, and patients with 4 or more PRLs had more aggressive disease, and experienced greater motor and cognitive disability at an earlier age [97, 98]. Also, PRLs have been detected in early RRMS [99]. PRLs represent MRI biomarkers that may reflect more compartmentalized inflammation [97] as they can be tracked over time and have promise in clinical trials to assess treatment response [100].

Slowly expanding lesions (SELs) are another proposed biomarker of chronic lesion activity [101]. Although SELs are more frequently thought to be associated with progressive MS [102], they have been reported to develop in 92% of patients with early RRMS [101]. Moreover, a higher number, volume and relative proportion of SELs—and in combination with PRLs – have been associated with a higher risk of disability progression and conversion to SPMS when identified in patients with early RRMS [101, 103]. In fact, SELs co-localized with PRLs, which exhibit severe accumulation of active tissue damage over time, may represent the most destructive type of chronic MS lesion [104]. As such, the presence of SELs and PRLs could serve as a biomarker and predictor of more severe disease activity early in the disease course [101, 103]. However, PRL validation and reliability, standardization of MRI methods, and clinical training of neurologists and neuroradiologists are required before PRLs can be implemented in clinical practice [105]. It is likely that additional technical requirements will also apply to the use of SELs in clinical practice.

MRI can also be used to detect veins located in the center of white matter lesions; this “central vein sign” can predict a diagnosis of MS in patients with otherwise atypical features of MS. With further research, this potential biomarker could facilitate diagnosis in clinical practice [106].

Both PRLs and the central vein sign appear to be specific for MS and have shown specificity for MS comparable and in some cases greater than OCBs: one study of scans from 112 patients with CIS and 35 in a non-MS group taken in routine clinical assessments found that the presence of ≥ 3 lesions with central vein signs or one PRL had high sensitivity (70%) and specificity (86%) for predicting conversion from CIS to MS [107]. Similarly, a retrospective study of 412 cases revealed that central vein signs could discriminate MS from non-MS cases with 99% sensitivity and 96% specificity; a combination of ≥ 1 PRL and ≥ 40% central vein signs gave a sensitivity of 59% and specificity of 99% [108]. Used in combination, these markers could be highly useful in the differential diagnosis of MS, but more prospective studies are required to validate PRLs and central vein signs as diagnostic biomarkers [108].

## Disease-modifying therapies (DMTs)

### Clinical guidelines on the use of disease-modifying therapies

Early treatment with DMTs is recommended for those patients with active RRMS or CIS, as defined by clinical relapses and/or MRI activity (active lesions, new or enlarging T2 lesions) [11]. In many cases, HETs are reserved for patients with highly active MS, which may be based on relapses and MRI activity [7], although it is becoming an increasingly common practice in certain MS centers and clinical practices to use HET even in the absence of classical markers of highly active disease [109, 110]. Current MS guidelines do not include management of RIS [7, 11]; however, a recent clinical trial showed that DMT use in patients with RIS could significantly reduce the risk of a first clinical demyelinating event and reduce the number of MRI lesions [111], which supports early treatment in the spectrum of demyelinating disease [111]. Furthermore, a recent observational study of 580 patients with a first demyelinating event reported an association between early treatment and a reduction in the long-term risk of disability accrual [112]. The results from other ongoing clinical trials investigating DMTs in patients with RIS could inform the treatment of these patients [113, 114].

### Early initiation of HET

Currently approved HETs include alemtuzumab, cladribine, natalizumab, ocrelizumab, ofatumumab, and ublituximab [115, 116]; opinion is mixed on the exact definition of HETs and whether sphingosine-1-phosphate modulators (fingolimod, siponimod, ozanimod, and ponesimod) should be included (Table 3) [117].

**Table 3:** Studies on early HET vs. MET and early vs. delayed initiation HET

Study	Design	Patients	HETs	Comparators	Outcomes
Early HET vs. early MET (with/without escalation)					
Labiano-Fontcuberta et al. (2022) [118]	Prospective longitudinal study	MS (*N* = 695)	Alemtuzumab, cyclophosphamide, mitoxantrone, natalizumab, ocrelizumab, ofatumumab, rituximab	Cladribine, dimethyl fumarate, fingolimod, glatiramer acetate, interferons, teriflunomide	Delay in starting HET was associated with cognitive worsening. MET was associated with higher risk of cognitive worsening vs. early HET
He et al. (2020) [119]	Observational cohort study	RRMS (*N* = 544)	Alemtuzumab, mitoxantrone, natalizumab, ocrelizumab, rituximab	Cladribine, dimethyl fumarate, fingolimod, glatiramer acetate, interferon beta, teriflunomide	Early HET (< 2 years after disease onset) was associated with lower long-term disability and lower hazard of disability progression vs. late HET (4–6 years after disease onset)
Simonsen et al. (2021) [10]	Real-world cohort study	MS (*N* = 694)	Alemtuzumab, fingolimod, natalizumab	Dimethyl fumarate, glatiramer acetate, interferons, teriflunomide	Patients starting HET were more likely to achieve NEDA at 1 and 2 years vs. patients starting MET
Harding et al. (2019) [120]	Real-world cohort study	MS (*N* = 592)	Alemtuzumab, natalizumab	Dimethyl fumarate, fingolimod, glatiramer acetate, interferons, teriflunomide	Mean change in EDSS score at 5 years was lower with HET vs. MET
Buron et al. (2020) [121]	Observational cohort study	RRMS (*N* = 388)	Alemtuzumab, cladribine, daclizumab, fingolimod, natalizumab, ocrelizumab	Dimethyl fumarate, glatiramer acetate, interferon beta, teriflunomide	At 4 years: 47% lower rate of EDSS score worsening and 50% lower rate of first relapse with HET vs. MET
Brown et al. (2019) [122]	Observational cohort study	RRMS (*N* = 1555)	Alemtuzumab, fingolimod, natalizumab	Glatiramer acetate, interferon beta	Lower risk (HR, 0.66) of conversion to SPMS with HET vs. MET over a median of 5.8 years
Iaffaldano et al. (2021) [123]	Observational cohort study	RRMS (*N* = 2702)	Alemtuzumab, cladribine, fingolimod, natalizumab, ocrelizumab	Azathioprine, dimethyl fumarate, glatiramer acetate, interferon beta, teriflunomide	Change in EDSS score was lower with HET vs. MET for up to 10 years
Rojas et al. (2022) [124]	Retrospective cohort study	MS (*N* = 305)	Alemtuzumab, cladribine, mitoxantrone, natalizumab, ocrelizumab, rituximab	Dimethyl fumarate, fingolimod, glatiramer acetate, interferon beta, teriflunomide	HET decreased risk of EDSS score progression, relapses, and new MRI activity vs. MET
Early initiation HET vs. delayed initiation HET					
Labiano-Fontcuberta et al. (2022) [118]	Prospective longitudinal study	MS (*N* = 695)	Alemtuzumab, cyclophosphamide, mitoxantrone, natalizumab, ocrelizumab, ofatumumab, rituximab	N/A	Each year of delay in starting HET was associated with cognitive worsening at 12 months (OR, 1.03)
Merkel et al. (2017) [125]	Systematic review	RRMS (12 studies)	Alemtuzumab, fingolimod, natalizumab	N/A	Early HET offers improved control of relapse activity vs. delayed HET

*EDSS* Expanded Disability Status Scale, *HET* high-efficacy therapy, *HR* hazard ratio, *MET* moderate-efficacy therapy, *MRI* magnetic resonance imaging, *MS* multiple sclerosis, *N/A* not applicable, *NEDA* no evidence of disease activity, *OR* odds ratio, *RRMS* relapsing–remitting multiple sclerosis, *SPMS* secondary progressive multiple sclerosis

A considerable body of evidence exists showing improved outcomes for patients with MS with early HET versus early medium-efficacy therapy and for early versus delayed use of HET, including delays in cognitive worsening and reductions in disability progression, relapses rates, and MRI activity (Table 3) [10, 118–125]. Moreover, the window of opportunity for optimum benefit from treating MS with HET is in the early stages of disease and at younger age [126]; a meta-analysis of 38 studies in > 28,000 subjects demonstrated an advantage for HET over low-efficacy therapy in early MS, before ~ 40 years of age [127]. The detection of PIRA and “silent progression” in patients with early MS, along with clinical evidence of HETs preventing disability accumulation regardless of relapse, further supports the use of early HET [41, 43, 128, 129].

Two randomized clinical trials, Traditional Versus Early Aggressive Therapy for Multiple Sclerosis (TREAT-MS; NCT03500328) and Determining the Effectiveness of early Intensive Versus Escalation Approaches for RRMS (DELIVER-MS, NCT03535298), are currently underway to examine the utility of early HET [130, 131]. With an estimated completion date of August 2025, TREAT-MS is a pragmatic controlled trial to evaluate whether early HET (natalizumab, alemtuzumab, ocrelizumab, rituximab, cladribine, or ofatumumab) versus traditional first-line therapy affects disability risk, and the effects on disability risk of switching to HET after breakthrough disease [131]. DELIVER-MS, with an estimated completion date of September 2026, is investigating whether early treatment with HET (alemtuzumab, natalizumab, rituximab, or ocrelizumab) improves prognosis by measuring brain atrophy over 3 years [130, 132]. It is anticipated that these trials will help guide treatment paradigms with existing and new therapies and support treatment decision making [130]. Furthermore, these trials could provide insights into brain atrophy, cognitive function, and patient-reported outcomes with early HET.

Although HET initiation early in the disease course may improve prognosis, several barriers to early HET versus escalation therapy exist. Fewer than 25% of patients diagnosed with MS received HET as their first-line treatment in Europe in 2019; HET is also substantially underused in the United States [115, 133]. A current lack of long-term randomized controlled trials informing on this approach means that payers and physicians could be reticent in using early HET until robust evidence exists. As such, national and regional guidelines still recommend starting treatment with less-effective therapy and restrict HET to later stages or after treatment failure; these delays can be due to reimbursement rather than regulatory criteria [115, 133]. Similarly, insurance companies can restrict access to certain DMTs, including HETs, despite their regulatory approval [134]. Among physicians, hesitancy in prescribing HETs may be due to safety concerns associated with more potent anti-inflammatory agents as well as reduced urgency and consideration for factors associated with poor prognosis in MS [115, 133]. In addition, the management of HET initiation and monitoring can present a challenge [135].

The benefit–risk profile for early use of HET should be considered in individual patients with an MS diagnosis and weighed against the risk of MS disease progression; decision-making needs to consider risks associated with specific HETs [115]. Therefore, patients need to be involved in treatment decisions so their preferences and concerns are addressed [7], because patients and physicians may not have the same perceptions of treatment risk [136]. In addition, it is anticipated that DELIVER-MS and TREAT-MS will shed further light on the evolving paradigm of HET for early MS in the real-world setting.

The accumulating evidence in support of early HET creates an urgency that may put pressure on neurologists to diagnose MS early, which could increase the risk of misdiagnosis if the McDonald criteria have not been correctly applied [66]. It is therefore paramount that an accurate diagnosis is made in line with the McDonald criteria to ensure optimal outcomes for patients [66, 137].

### Disease activity after DMT initiation

After initiation of DMTs, patients may be classified based on the presence of relapses and MRI lesion activity. However, phase 3 clinical trials have revealed a considerable proportion of patients with PIRA after treatment with DMTs [43, 128]. In addition, an observational study in 480 patients with CIS or RRMS found that long-term disease evolution to SPMS occurred independently of relapses and new MRI lesions [129]. Hidden indicators of MS, such as cognitive impairment, depression, anxiety, fatigue, and sleep disturbance, did not significantly improve with DMT use in an observational study of 440 patients [138]. Patients with MS have reported that these hidden indicators are not sufficiently prioritized and addressed with currently available therapies; thus, a more comprehensive treatment approach is required [139]. Taken together, these observations highlight the need to more fully assess the effectiveness of existing DMTs in mitigating factors of disease beyond relapses and lesion activity.

In addition to their use in early disease, molecular biomarkers could help monitor disease progression after DMT initiation in the absence of relapses or MRI lesion activity. The CMSC has proposed the use of CSF and serum NfL in determining response to therapy at an individual level [84]. In addition, NfL could be used to measure disease activity beyond that detected with clinical and MRI markers, and as a biomarker to monitor treatment effectiveness (Table 2). However, more evidence is needed to determine the role of molecular biomarkers in the decision to escalate treatment from a traditional therapy to a HET.

No evidence of disease activity (NEDA) is a marker used in clinical trials to measure disease activity and treatment response; NEDA-3 is a composite of no relapses, no disability progression, and no MRI lesion activity [140]. However, focusing solely on these measures risks missing underlying neurodegeneration and brain atrophy, often termed “silent progression” [129]. In one study of 42 patients with MS, although 31% achieved NEDA-3 after 2 years, 58% of those with NEDA-3 still developed cognitive worsening [141]. In addition, a longitudinal registry study found that NEDA-3 at 2 years did not predict long-term disease stability [142]. Consequently, a more stringent NEDA-4 is used, which includes additional measures of disease activity, such as brain atrophy [143], NfL [144, 145], or Symbol Digit Modalities Test (SDMT) [146]. Multiple studies with DMTs have demonstrated the greater stringency of NEDA-4 over NEDA-3; a pooled analysis of clinical trials found that 31.0% of 783 fingolimod-treated patients achieved NEDA-3, whereas only 19.7% of 706 patients achieved NEDA-4 (NEDA-3 plus brain atrophy) [143]. Similarly, a phase 3 trial of ponesimod reported 25.0% of patients achieved NEDA-3, but only 11.4% achieved NEDA-4 (NEDA-3 plus brain atrophy) [147]. In an observational study, 58% of 48 patients achieved NEDA-3, whereas only 29% achieved NEDA-4 (NEDA-3 plus brain atrophy) [148]. Another observational study of 45 patients reported 1-year NEDA rates of 60% for NEDA-3, 38% for NEDA-4 (NEDA-3 plus brain atrophy), and 53% for NEDA-4 (NEDA-3 plus SDMT) [146]. An expanded definition of NEDA (NEDA-5) that includes cognition, brain atrophy, inflammatory, and axonal damage markers has been proposed [149]. This would further lower the threshold to detect potentially clinically meaningful changes in patients, to promote urgency in clinical decision-making for patients treated suboptimally.

## Consensus guidelines for identifying and managing early disease activity: an unmet need

Greater emphasis on “hidden” symptoms of early MS, such as cognitive and other neuropsychiatric changes, allows disability accrual to be measured more completely and warrants further consideration for future diagnostic and monitoring guidelines. Validated assessment tools for cognitive testing, combined with unified criteria for identifying cognitive impairment, would enable this approach. Electronic self-administered tools such as the Processing Speed Test are potentially practical and convenient ways of testing cognition. In the near future, as validated assays and data demonstrating actionable thresholds become available, biomarkers such as NfL could be used in real-world clinical practice for prognosis alongside clinical evaluations and imaging [84].

In the future, new consensus will likely be needed on early use of HETs as new data on “hidden” symptoms become available. The 2017 update of the McDonald criteria advises caution and postponement of a definitive MS diagnosis and delayed initiation of long-term DMTs in cases presenting atypical features or in the absence of typical CIS, to avoid misdiagnosis [13]. This highlights the importance of diagnostic tools and markers in atypical cases to enable prompt and accurate diagnosis, and therefore early initiation of therapy.

Judicious guidelines on starting therapy that take prognostic factors into consideration are required to ensure patients receive effective therapy and provide budget certainty to payers [7, 115]. Any concerns around initiating early HETs must be weighed against the risk to patients of disease progression and irreversible brain damage through missed opportunities of early effective treatment [10, 115]. A comprehensive therapeutic algorithm for treatment decisions may be required to improve access to HET [115]; this could integrate a range of prognostic factors to enable better risk stratification [115, 150].

## Conclusions

Diagnosis and treatment of MS at the earliest possible stages are critical for optimal outcomes. Although current guidelines define disease activity primarily in terms of clinical relapses and MRI lesion activity, these measures do not necessarily capture early pathological changes in MS. Impaired cognition, fatigue, brain atrophy, and elevated NfL levels are evident early in the disease process and may be used as indicators of poor prognosis. Therefore, these should be considered when defining disease activity, to provide a more integrated and comprehensive understanding of MS that reflects underlying neurodegeneration, other disease signs, and patient experiences.

Early detection of MS allows early treatment initiation. In light of emerging evidence and pending outcomes of ongoing clinical trials, early HET is appropriate and can be considered not only in cases of severe disease activity. Long-term safety data and clinical trial data on early HET are needed to help give physicians and payers confidence in its use. As new data from ongoing clinical trials become available, the current management guidelines will require updating to provide additional support to physicians with regard to early initiation of HET.

## Data Availability

Not applicable.
